# The main component of an alarm pheromone of kissing bugs plays multiple roles in the cognitive modulation of the escape response

**DOI:** 10.3389/fnbeh.2013.00077

**Published:** 2013-07-05

**Authors:** Sebastian Minoli, Florencia Palottini, Gabriel Manrique

**Affiliations:** Laboratorio de Fisiología de Insectos, Departamento de Biodiversidad y Biología Experimental, Facultad de Ciencias Exactas y Naturales, Universidad de Buenos Aires, IBBEA, CONICET-UBABuenos Aires, Argentina

**Keywords:** learning, alarm-pheromone, plasticity, triatomines, associative, non-associative

## Abstract

Innate responses in animals can be modulated by experience. Disturbed adults of the triatomine bug *Triatoma infestans* release an alarm pheromone (AP) that elicits an escape response in conspecific larvae. The main component of this AP, the isobutyric acid (IsoAc), alone has already shown to generate an escape response in this species. However, not much is known about the modulation of this behavior by non-associative and associative cognitive processes. We present here evidences of the cognitive capacities of *T. infestans* larvae in an escape context under different conditioning paradigms, including IsoAc in different roles. We show that: (1) the duration of a pre-exposure to IsoAc plays a main role in determining the type of non-associative learning expressed: short time pre-exposures elicit a sensitization while a longer pre-exposure time triggers a switch from repellence to attractiveness; (2) a simple pre-exposure event is enough to modulate the escape response of larvae to the AP and to its main component: IsoAc; (3) IsoAc and the AP are perceived as different chemical entities; (4) an association between IsoAc and an aversive stimulus can be created under a classical conditioning paradigm; (5) an association between IsoAc and a self-action can be generated under an operant conditioning. These results evince that IsoAc can attain multiple and different cognitive roles in the modulation of the escape response of triatomines and show how cognitive processes can modulate a key behavior for surviving, as it is the escaping response in presence of a potential danger in insects.

## Introduction

Chemical communication in insects is one of the main ways to find sexual partner, aggregate or prevent conspecifics from a danger, among other behaviors. In triatomine bugs (Hemiptera, Reduviidae, Triatominae), adults bear paired exocrine glands in the thorax (metasternal gland) and in the abdomen (Brindley's gland), which are absent in larvae (Brindley, 1930; Schofield and Upton, 1978). It has been proposed for different triatomine species that metasternal gland volatiles mediate sexual communication between adults (Manrique et al., 2006; Crespo and Manrique, 2007; Pontes et al., 2008; Vitta et al., 2009; Zacharias et al., 2010; Manrique and Lorenzo, 2012; Pontes and Lorenzo, 2012). The Brindley's glands, which secrete isobutyric acid (IsoAc) as their main component, are likely to be associated with alarm and defense functions (Kälin and Barrett, 1975; Schofield, 1979; Ward, 1981; Cruz-López et al., 1995; Rojas et al., 2002; Manrique et al., 2006). Particularly in the hematophagous bug *Triatoma infestans* Klug 1834, the existence of an alarm pheromone (AP) released by disturbed adults that elicits an escape response of conspecific larvae has been reported (Manrique et al., 2006). Moreover, IsoAc alone has also shown to modulate the behavior of this species, being attractive or repellent according to the presented dose (Ward, 1981; Guerenstein and Guerin, 2001).

Although innate responses are essential for surviving, cognitive processes confer individuals the aptitude to acquire or improve skills after a first experience. This capacity varies across species, individuals, and even throughout lifespan and can be modulated by several features of training procedures. Sensitization and habituation are cognitive processes expressed in almost all animals, evincing the adaptive value of these simple forms of plasticity of behavior. Both processes involve non-associative conditionings, which cause a change in behavior as a result of a first sensorial experience which is not associated to any other cue or reward. While sensitization helps to increase attention to a particular cue (Monteith, 1963; Rakitin et al., 1991; Braun and Bicker, 1992), increasing the probability to find it or avoid it (Rakitin et al., 1991; Hammer et al., 1994; Aggio et al., 1996; Walters et al., 2001; Anderson et al., 2003, 2007; Grubb and Thompson, 2004; Anton et al., 2011; Guerrieri et al., 2012; Minoli et al., 2012), habituation helps to filter out information which is no longer relevant (Duerr and Quinn, 1982). Generally, short exposure times lead mostly to sensitization processes while long exposures provoke an habituation of the response.

Associative learning is the process by which an association between two stimuli or a behavior and a stimulus is consolidated, if properly reinforced (Bitterman et al., 1983; Heisenberg et al., 1985; Menzel and Muller, 1996). Two main forms of associative learning have been described in animals. In Pavlov's classical conditioning (Pavlov, 1927) a previously neutral stimulus is repeatedly presented together with a reflex eliciting stimuli followed by a reinforcement, until eventually the neutral stimulus will elicit a response on its own. In Skinner's operant conditioning (Skinner, 1937) a certain behavior is followed by a reinforcement, resulting in an altered probability that the behavior will happen again.

Although learning and memory have been widely studied in many insect species, very little has been done to describe the cognitive abilities of hematophagous insects. Host preference or oviposition-sites fidelity of mosquitoes have been shown to be modulated by experience (McCall and Eaton, 2001; McCall et al., 2001; McCall and Kelly, 2002; Alonso et al., 2003; Kaur et al., 2003). However, recent work has been published in which the cognitive capacities of triatomines are evinced. In these works, the authors show that under a classical paradigm, *Rhodnius prolixus* is able to associate a neutral odor with a positive (Vinauger et al., 2011a) and a negative reinforcement (Vinauger et al., 2011b). Moreover, after a first experience, this species learned to avoid host odors negatively reinforced (Vinauger et al., 2012).

In this work we analyzed the experience-dependent modulation of the escape response of *T. infestans* larvae when confronted to the AP or to its main component, IsoAc. Non-associative protocols were applied to analyze if a chemical pre-exposure may change the escape response of larvae confronted to the AP or the IsoAc. Short and long pre-exposure times were applied to investigate if pre-exposure duration may account for the switch from a sensitization to an habituation process. Crossed pre-exposure and tests with AP and/or IsoAc were carried out to find out if they are perceived as different chemical entities or not. Classical conditioning of the escape response was studied pairing the IsoAc with a mechanical aversive stimulus and then measuring changes in the response to IsoAc. Operant conditioning was analyzed in a spatial preference paradigm by training individuals to avoid one zone of an experimental arena by delivering IsoAc every time they entered such zone and measuring then changes in their spatial preference caused by this training. We present clear evidences of the cognitive modulation of the escape response in triatomines under all forms of learning tested. We discuss the multiple modulatory roles of the main component of the AP, IsoAc, in different cognitive processes.

## Materials and methods

Larvae of *T. infestans* were reared in the laboratory insectary at 28 ± 2°C temperature and 60 ± 20% relative humidity, under a 12:12 h L/D illumination regime. All instars were fed weekly on live hens handled according to the biosafety rules from the *Servicio de Higiene y Seguridad of the Facultad de Ciencias Exactas y Naturales, Universidad de Buenos Aires*. For the assays, 15–25 days old non-fed fourth instar larvae were used. A total of 1600 larvae were used along this work. Insects were used only once and then discarded.

All experiments (training and test procedures, see below) were carried out during the first hours of the scotophase (i.e., 1–5 h after lights were turned-off) as to match the maximal activity period observed for these insects (Lazzari, 1992). Experiments were performed under total darkness conditions to match the phase of photoperiod in which animals were tested (i.e., scotophase) and at the same time to avoid the possible utilization of visual cues by larvae. The temperature of the experimental room was set to 25 ± 1°C before the beginning of each assay with an electric fan heater, which was turned-off before the start of the experiments. The room's relative humidity range was 40 ± 10%.

## Experiment 1. Non-associative learning: Chemical pre-exposure

We applied different pre-exposure protocols using different odors and times, and we analyzed if this chemical stimulation could modify the escape response of larvae of *T. infestans* confronted to the same or different odors and/or doses.

### Training procedures

**Figure 1:**
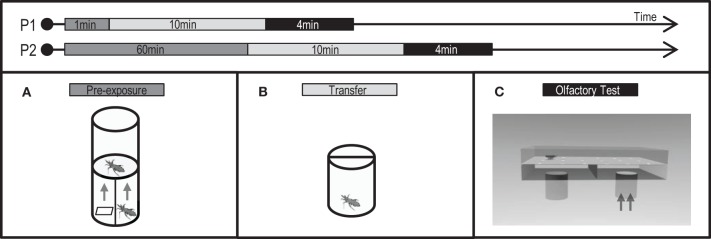
**Protocols (P1: short pre-exposure, P2: long pre-exposure) applied and experimental devices used to analyze the effects of a non-associative exposure in the escape response of *T. infestans* larvae**. Chemical pre-exposure was made stimulating with the alarm pheromone (AP) or with its main component, isobutyric acid (IsoAc). Pre-exposure flask **(A)**, transfer flask **(B)**, and test arena **(C)**.

Pre-exposure to different chemical stimuli was carried out using an acrylic cylindrical flask (Figure 1A, 6 cm height, 2.5 cm diameter) divided horizontally by a plastic mesh (0.5 mm pore). Larvae were placed over the mesh and the different stimuli sources at the bottom of the flask. In this way, individuals were exposed to the volatiles released by either one disturbed adult (AP) or a rectangular piece of filter paper (2 × 1 cm) loaded with 10 μg of IsoAc (i.e., approximate content of Brindley's glands, Palottini, pers. communication).

Pre-exposure to IsoAc was achieved either for a short (1 min, Figure 1: P1) or a long period (60 min, Figure 1: P2). For the short pre-exposure, 50 μl of dichloromethane (DCM) containing 10 μg of IsoAc were loaded on the filter piece of paper and inserted at the bottom of the pre-exposure flask. One larva was then placed over the plastic mesh for 1 min. For the long pre-exposure a similar procedure was performed but larvae were left over the mesh during 60 min. The stimulus-loaded filter paper (i.e., 50 μl of DCM with 10 μg of IsoAc) was changed for a new reloaded one every 15 min (i.e., four equally loaded pieces of paper) to ensure the presence of the odor in the air along time. Control assays were carried out by loading the solvent alone (i.e., 50 μl of DCM) in the filter paper (naive insects).

Pre-exposure to the AP was carried out by placing one adult in the lower part of the flask. Insects were allowed to climb onto a piece of filter paper, which was subsequently placed into the pre-exposure flask to avoid disturbing them. Once inside the flask, the stimulus-adult was artificially disturbed by grabbing one of its legs with forceps during 30 s. One larva was placed over the plastic mesh during 1 min of pre-exposure to the released blend. Control assays were carried out by leaving the adult undisturbed (naive insects).

After pre-exposure protocols, animals were transferred to individual acrylic cylindrical flasks (3 × 2.5 cm) (Figure 1B) in darkness for 10 min before the beginning of the tests in a dual-choice olfactometer (Figure 1C, see below).

### Measurement of the escape response in a dual-choice olfactometer

The escape response elicited by the AP of *T. infestans* adults or by IsoAc was tested using a dual-choice walking olfactometer in absence of air currents (Figure 1C). The arena consisted of a rectangular acrylic box (15 × 10 × 4 cm) with holes on the floor (2 mm diameter), attached to a sub-chamber divided in two equal parts by an odor-impermeable transversal acrylic plate, interconnected to independent lower openings into which removable flasks (10 ml) containing the stimuli were attached.

Either an undisturbed or a disturbed *T. infestans* adult or different doses of IsoAc (0.1, 10, or 1000 μg) in 50 μl of DCM, or 50 μl of DCM alone loaded on a piece of filter paper (2.5 × 0.5 cm) were placed in the stimulus-flasks. In this way, stimuli (the volatiles emitted by disturbed or undisturbed adults or by the loaded piece of paper) entered by diffusion into each of the two parts of the sub-chamber independently and continued to diffuse up to the arena through the holes, creating a chemical gradient over it. A Kraft paper with holes matching the floor holes served as substrate for the experimental larvae and avoided possible chemical contamination between assays, as it was changed every time a new bug was released.

In each individual assay, one flask was used as control (either an undisturbed adult or a DCM loaded filter paper was inserted) and the other was set as stimulus (i.e., a disturbed adult or IsoAc was inserted). Both flasks were attached to the sub-chamber and one larva was then placed in the middle of the arena and left covered with an inversed flask over it during 1 min for context familiarization and odor diffusion. The larva was then released by gently lifting the covering flask and its behavior was registered during 4 min by means of a video-camera connected to a digital recorder. Control assays were performed adding only DCM into both flasks. The position of the stimulus was randomly alternated between assays.

The time spent in each side of the arena was registered as a measure of the olfactory preference of insects. A preference index (PI) ranging from −1 to 1 was calculated as *PI* = (*T*_*C*_ − *T*_*S*_)/(*T*_*C*_ + *T*_*S*_), where *T*_*C*_ is the time (in seconds) spent in the control side of the arena and *T*_*S*_ the time spent in the side where the stimulus was added. In control series (i.e., no stimulus added) *T*_*S*_ is the time in seconds spent in one side of the arena chosen randomly. *PI*s near −1, 0, or 1 indicate repellence, random distribution, or attraction to the added stimulus, respectively. Deviations from a random distribution (i.e., *PI* = 0) of the larvae over the arena were assessed by means of One Sample *T*-Tests. Differences between pre-exposures were assessed by means of One Way ANOVAs followed by Tuckey *post-hoc* comparisons when needed. A total of 40 replicates were achieved for each treatment.

### Results 1A: Modulation of the escape response to IsoAc after a short or a long pre-exposure

**Figure 2:**
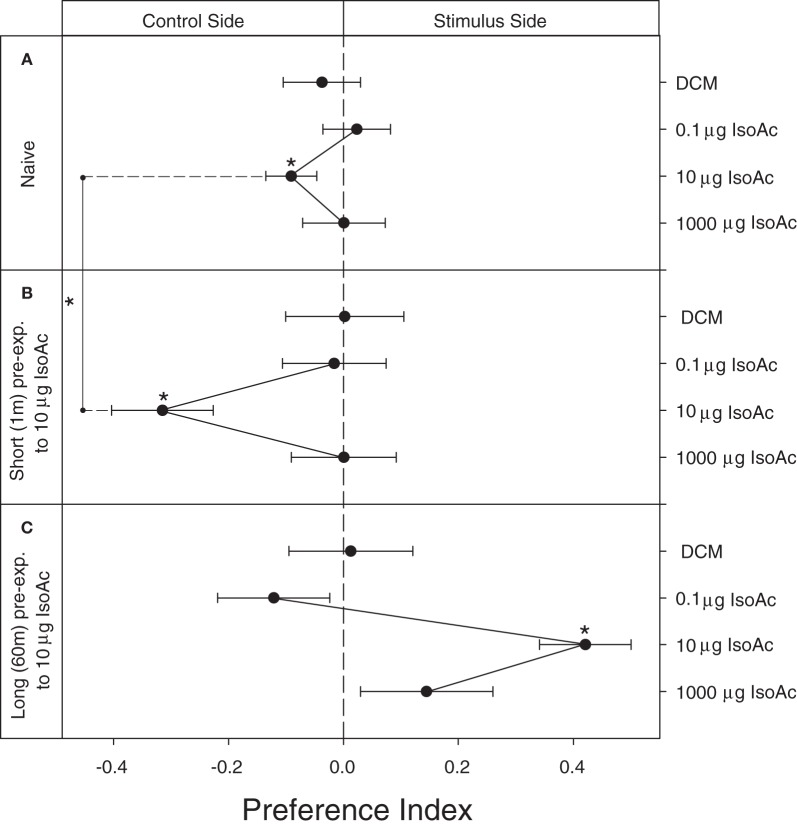
**Experiment 1A: Modulation of the escape response to IsoAc after a non-associative pre-exposure to the same compound. (A)** Naive, **(B)** short pre-exposed (1 m), and **(C)** long pre-exposed (60 m) larvae. *N* = 40 assays for each dose. Asterisks show significant differences from a *PI* = 0 (One Sample *T*-Test, *p* < 0.05). Asterisk over the vertical line show differences between treatments (*T*-Test, *p* < 0.05). Naive larvae escaped from 10 μg of IsoAc **(A)**. A clear sensitization effect after a short pre-exposure was evident **(B)**. A long pre-exposure protocol inversed the significance of the IsoAc **(C)**.

The main component of the AP of adult *T. infestans*, IsoAc, has already shown to repel adults of the same species (Ward, 1981). Here, we show that naive larvae are repelled by 10 μg of IsoAc (Figure 2A; One Sample *T*-Test, *p* = 0.04) and not by other doses (*p* > 0.05 for all cases). A similar but sharper dose-dependent avoidance was obtained after a short pre-exposure of 1 min to 10 μg of IsoAc (Figure 2B; One Sample *T*-Test, *p* = 0.001 for 10 μg IsoAc, *p* > 0.05 for other doses). A long exposure (60 m) to IsoAc resulted in a dramatic change from repellence to attractiveness to 10 μg of IsoAc (Figure 2C; One Sample *T*-Test, *p* = 0.00005).

To quantify the effect of pre-exposure we compared the intensity of the escape response of naive and short pre-exposed larvae elicited by 10 μg of IsoAc. We found a significantly higher repellence after a short pre-exposure to IsoAc (Figures 2A,B; *T*-Test, *p* = 0.0002; comparison showed with a vertical line).

### Results 1B: Cognitive discrimination between AP and IsoAc

**Figure 3:**
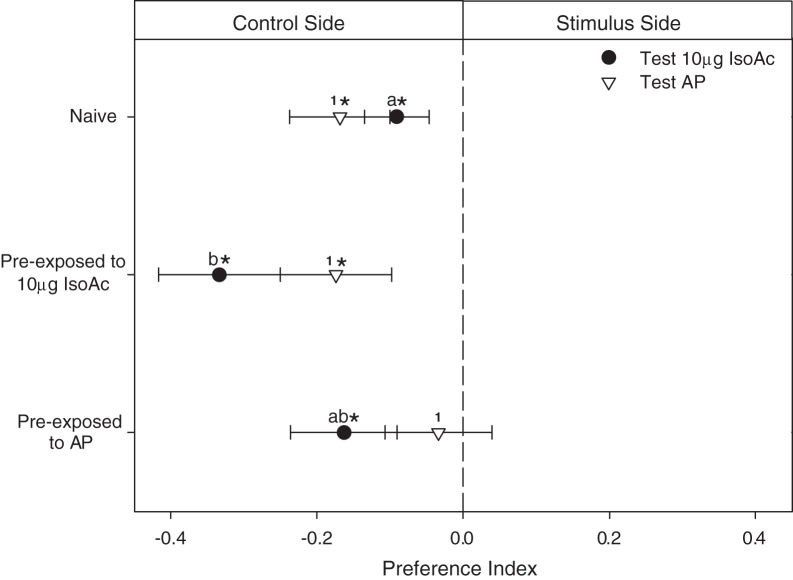
**Experiment 1B: Modulation of the escape response of larvae to IsoAc or to the AP after a non-associative pre-exposure to IsoAc or to the AP**. *N* = 40 assays for each treatment. Asterisks show significant differences from a *PI* = 0 (One Sample *T*-Test, *p* < 0.05). Different letters or numbers show significant differences between treatments (*T*-Test, *p* < 0.05). Naive larvae avoided both, 10 μg of IsoAc and the AP. Pre-exposure to IsoAc increased the escape response to the same compound but not to the AP. Pre-exposure to the AP did not change the innate escape response to IsoAc nor to AP.

We analyze here the lineal- and cross-effects of a brief chemical pre-exposure to the AP (i.e., a blend released by disturbed adults) or to its main component alone (IsoAc) in the escape response generated by the same two odors. As expected, all groups of larvae avoided the side of the arena containing 10 μg of IsoAc (Figure 3, black dots; One Sample *T*-Test, *p* < 0.05, for three cases). Similarly, naive- and IsoAc-exposed larvae avoided the AP (Figure 3, white triangles; One Sample *T*-Test, *p* < 0.05, for both cases). Only the larvae pre-exposed to AP did not avoid the AP during test (One Sample *T*-Test, *p* > 0.05).

When we compared the effect of pre-exposure, we found that the escape response to the AP did not vary with a chemical experience (Figure 3; white triangles; One Way ANOVA, *p* > 0.05, significant differences shown with different numbers). Conversely, a significant effect of pre-exposure to IsoAc over the escape response of larvae was revealed (black dots; One Way ANOVA, *p* = 0.04). *Post-hoc* comparisons showed that a brief pre-exposure to 10 μg of IsoAc increased the escape response to the same compound as compared to naive larvae (Tuckey, *p* = 0.03, significant differences shown with different letters) but a pre-exposure to AP did not (Tuckey, *p* > 0.05).

## Experiment 2. Associative learning: Classical conditioning

A classical conditioning arrangement was designed for *T. infestans* larvae in which the negative hedonic value of IsoAc was expected to increase by associating the presence of this aversive compound with a mechanical disturbance. In posterior tests, larvae should increase their avoidance behavior against IsoAc.

### Training procedures

**Figure 4:**
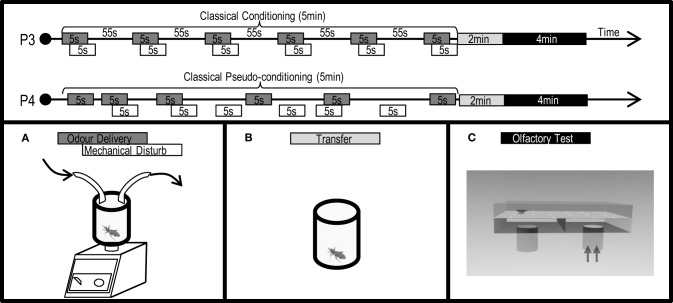
**Protocols (P3: conditioning, P4: pseudo-conditioning) applied and experimental devices used to analyze the effects of an associative classical conditioning in the escape response of *T. infestans* larvae promoted by isobutyric acid (IsoAc)**. Training flask **(A)**, transfer flask **(B)**, and test arena **(C)**.

Training was carried out by placing a larva inside a closed glass flask (Figure 4A, 5 cm height, 3 cm diameter) with one input-tube (5 mm diameter) bearing a clean air current, into which controlled doses of IsoAc could be added manually with an electronic switch by interposing a loaded piece of filter paper (10 or 1000 μg of IsoAc), and one output-tube conducting odors to an external extractor. The flask containing the larva was placed over a mixer (Vortex 1000 rpm) that generated a mechanical disturbance electronically controlled. The timing of the IsoAc delivery and the mechanical disturbance caused by the mixer was manipulated as to generate different learning protocols.

Conditioning assays (Figure 4: P3) consisted of 5 min trainings in which larvae perceived once every minute a trial, pairing a 5-s puff of IsoAc (10 or 1000 μg) with a 5-s mechanical disturbance, shifted 2 s forward (i.e., 6 trials, inter trial interval 53 s). A pseudo-conditioning protocol (Figure 4: P4) was performed in which 6 puffs of IsoAc and 6 mechanical disturbances were delivered in a random manner (i.e., not paired). Insects in the naive group were not submitted to IsoAc nor to the mechanical disturbance.

After training, animals were transferred to individual acrylic cylindrical flasks (3 × 2.5 cm) (Figure 4B) in darkness for 2 min before the beginning of the tests in a dual-choice olfactometer (Figure 4C).

### Measurement of the escape response in a dual-choice olfactometer

The escape response triggered by IsoAc was tested using the dual-choice walking olfactometer in absence of air currents described above (Figure 4C). Two doses of IsoAc were loaded on the filter paper matching the dose used during training: 10 or 1000 μg. For each test, the time spent in each side of the arena by each larva was registered during 4 min. The PI was calculated as described before (see Measurement of the Escape Response in a Dual-Choice Olfactometer, for more details). Deviations from a random distribution (i.e., *PI* = 0) were assessed for each treatment by means of One Sample *T*-Tests. A total of 40 replicates were achieved for each treatment.

### Results: Modulation of the escape response to IsoAc after a classical association with a mechanical disturbance

**Figure 5:**
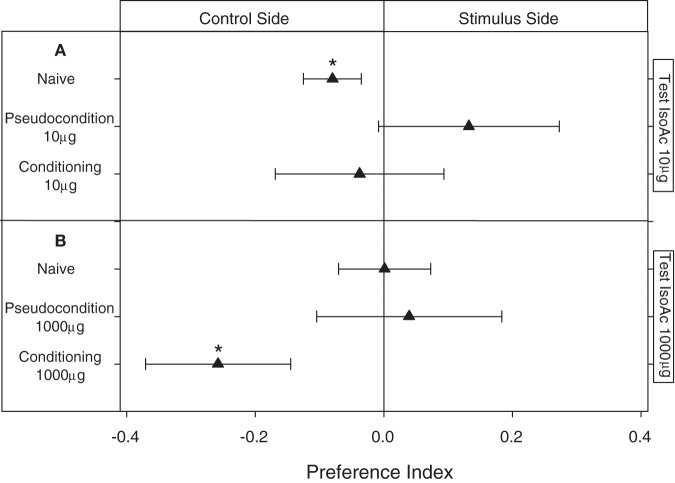
**Experiment 2: Modulation of the escape response of larvae to IsoAc after an associative classical conditioning training. (A)** Training and test with 10 μg of IsoAc. **(B)** Training and test with 1000 μg of IsoAc. *N* = 40 assays for each treatment. Asterisks show significant differences from a *PI* = 0 (One Sample *T*-Test, *p* < 0.05). No changes in the escape response were observed after an aversive conditioning with 10 μg of IsoAc **(A)**. However, although not-trained animals (naive or pseudo-conditioning groups) did not avoid 1000 μg of IsoAc, after an associative training they were strongly repelled by this dose **(B)**.

In these assays we applied a classical conditioning pairing the delivery of a puff of IsoAc with a mechanical disturbance. Naive larvae presented (as shown before) an innate escape response to 10 μg of IsoAc (Figure 5A; One Sample *T*-test, *p* = 0.04). Although this repellent effect was expected to increase by a training in which the hedonic value of IsoAc 10 μg was supposed to become more negative after an association with a second aversive stimulus (i.e., the mechanical disturbance), no escape response at all was observed for the conditioning group (Figure 5A; One Sample *T*-test, *p* > 0.05). As the pseudo-conditioning assays with 10 μg of IsoAc (in which the delivery of the puff of IsoAc was not paired in time with the mechanical disturbance) also resulted in a lack of response to 10 μg during tests (Figure 5A; One Sample *T*-test, *p* > 0.05), the vanishing of the escape response might then be an effect of habituation to the IsoAc delivered during training.

When 1000 μg of IsoAc was used during training, even if naive animals did not avoid the zone with this dose of IsoAc (Figure 5B; One Sample *T*-test, *p* > 0.05), once submitted to a conditioning in which we paired 1000 μg of IsoAc with the mechanical disturbance, larvae started to avoid it (Figure 5B; One Sample *T*-test, *p* = 0.02). In their corresponding pseudo-conditioning assays, no escape response was registered (Figure 5B; One Sample *T*-test, *p* > 0.05). These results show that the lack of escape response presented by larvae when 1000 μg of IsoAc was presented in one side of the arena (Experiment 1A, Figure 2) is not a constraint of the setup (e.g., due to a chemical homogenization of the arena caused by high doses), but instead a result of the insects' preference, as in this section larvae avoided the same dose.

## Experiment 3. Associative learning: Operant conditioning

An operant conditioning training was designed in which larvae of *T. infestans* were trained to avoid one half of an experimental arena (predetermined as *punished* side) by delivering IsoAc whenever they entered this zone during training. During posterior tests without odors delivered, larvae were expected to avoid the side where the IsoAc was delivered during training.

### Training procedures

**Figure 6:**
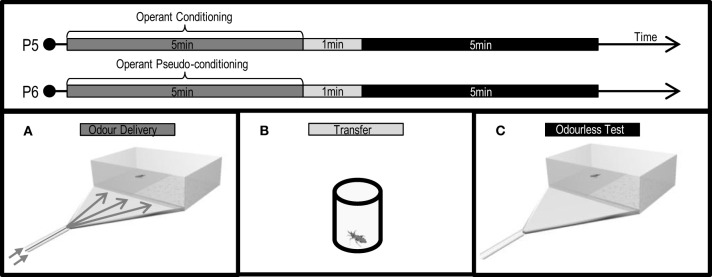
**Protocols (P5: conditioning, P6: pseudo-conditioning) applied and experimental devices used to analyze the effects of an associative operant conditioning in the escape response of *T. infestans* larvae promoted by isobutyric acid (IsoAc)**. Training arena **(A)**, transfer flask **(B)**, and test arena **(C)**.

Training was performed using a rectangular experimental arena (8 × 5 × 4 cm) with half of its floor covered with smooth paper and the other half with rough paper (Figure 6A). A silicon tube connected to a triangular flat diffusor delivered continuously either a clean (50 μl of DCM) or a IsoAc loaded air current to the whole arena, at the floor level. To deliver the IsoAc to the insects, 10 or 1000 μg of IsoAc in 50 μl of DCM were loaded on a filter paper (2.5 × 0.5 cm) and interposed in the clean air current. Before each assay, one side of the arena (and so one texture) was settled as the *punished* side and the other as the *safe* side in a pseudo-random manner (i.e., randomly but balanced along 40 replicates). The timing of the IsoAc delivery was defined by the position of each larva in conditioning assays (Figure 6: P5) or manipulated by the experimenter in pseudo-conditioning assays (Figure 6: P6).

For conditioning assays, one larva was released in the middle of the arena and, during 5 min of training, IsoAc was added to the current whenever the larva entered the previously determined *punished* side. Pseudo-conditioning assays were performed in which IsoAc was added to the current independently from the position of the larva over the arena during 5 min. The total IsoAc time delivered during pseudo-conditioning assays was calculated from conditioning series. Two doses of IsoAc were used during trainings: 10 or 1000 μg.

After training, animals were transferred to individual acrylic cylindrical flasks (3 × 2.5 cm) (Figure 6B) in darkness for 1 min before the beginning of the odorless tests (Figure 6C).

### Measurement of the spatial preference in an experimental arena without odors

The spatial preference (guided by the texture of the floor) was registered over the experimental arena used during training (Figure 6C) but without the addition of IsoAc to the air current (i.e., a continuous current bearing only 50 μl of DCM).

In each assay, one larva was placed in the middle of the arena covered with an inversed flask. After 1 min of context familiarization the cover was gently lifted releasing the larva. Its behavior was then registered using a video camera connected to a digital recorder.

The time spent in each side of the arena was registered during conditioning (5 min) and tests (5 min). A PI ranging from −1 to 1 was calculated as *PI* = (*T*_*S*_ – *T*_*P*_)/(*T*_*S*_ + *T*_*P*_), where T_S_ is the time (in seconds) spent in the *safe* side of the arena and *T*_*P*_ the time spent in the *punished* side. *PI*s near −1, 0, or 1 indicate preference to stay in the *punished* side, random distribution or preference to stay in the *safe* side of the arena, respectively. Deviations from a random distribution (i.e., *PI* = 0) of the larvae over the arena were assessed by means of One Sample *T*-Tests. A total of 40 replicates were achieved for each treatment.

### Results: Avoidance of a neutral zone after an operant association with IsoAc

**Figure 7:**
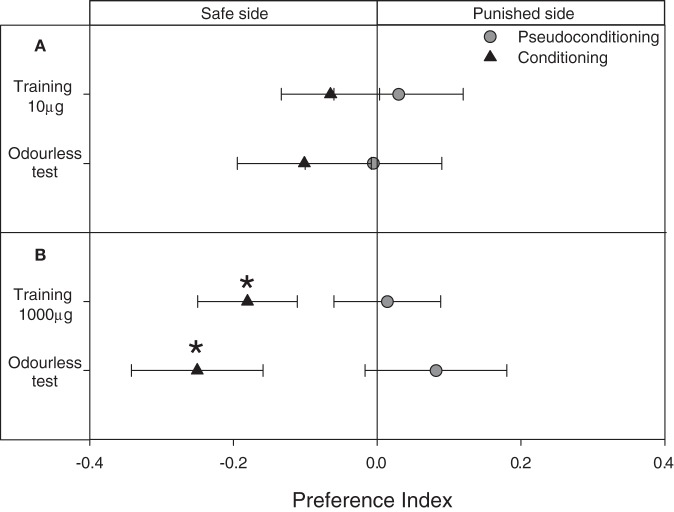
**Experiment 3: Modulation of the escape response of larvae to IsoAc after an associative operant conditioning training. (A)** Training with 10 μg of IsoAc. **(B)** Training with 1000 μg of IsoAc. *N* = 40 assays for each treatment. Asterisks show significant differences from a *PI* = 0 (One Sample *T*-Test, *p* < 0.05). A not-significant trend to avoid the *punished* side was observed when 10 μg IsoAc was used as punishment during training **(A)**. When 1000 μg of IsoAc were used during training, larvae learned to avoid the *punished* side and continued to avoid it during odorless tests **(B)**.

An operant conditioning paradigm was applied by delivering a punishment (puff with IsoAc) whenever larvae entered the *punished* side of the arena. Differently from previous experiments, this protocol allowed us to calculate the *PI*s already during trainings. We found that when 10 μg of IsoAc was used as negative reinforcement, although a trend to avoid the *punished* side was observed during both, training and test, no statistical differences were observed from a random distribution (Figure 7A; One Sample *T*-test, *p* > 0.05, for both cases). But, when 1000 μg of IsoAc were used as punishment, a significant repellence was observed already during training (Figure 7B; One Sample *T*-test, *p* = 0.01). After a transfer event of 1 min and even without odor-delivery during tests, larvae continued to avoid the side previously defined as *punished* (Figure 7B; One Sample *T*-test, *p* = 0.009).

Larvae stimulated with either 10 or 1000 μg of IsoAc independently from their position in the arena (i.e., pseudo-conditioning series) presented a random distribution during both, training and tests (Figures 7A,B; One Sample *T*-test, *p* > 0.05 for all cases).

## Discussion

All forms of learning tested here showed to modulate the escape response of *T. infestans* to IsoAc. Although a highly relevant behavior for survival (as it is escaping from a potential danger) generally requires a genetic basis, it was shown here that it is strongly modulated by experience in many ways. The sensitization elicited after a brief perception of the IsoAc was reflected in an increased attention to this compound and an increased proneness to respond. In natural environments, being more sensitive to an AP, if it has been recently and briefly perceived (i.e., meaning that the danger is probably still around), can be the cue to perform a rapid escape and so survive.

Although an habituation expressed as a lack of response was expected after a long pre-exposure to IsoAc, we found instead an inversion of the significance of the signal, becoming attractive for larvae. IsoAc has been already reported by other authors to be repellent at high doses and to become attractive at low doses (Ward, 1981; Guerenstein and Guerin, 2001). Besides being released by Brindley's glands, IsoAc is a constituent of human sweat (Cork and Park, 1996) and probably of other triatomine hosts. However, the amount of this compound release by vertebrate skin is much lower as compared to the AP content. It might happen then that the attraction of long-pre-exposed larvae to a normally repellent dose of IsoAc (10 μg) was a result of a sub-estimation of the real dose sensed due to an habituation process. Nevertheless, other alternative processes such as sensory priming might also explain the attraction after a long-term exposition (Schacter and Buckner, 1998).

We can discard that the results observed were a case of sensory adaptation after a pre-exposure to IsoAc, as we found either an increased or an inversed response to IsoAc, but never a lack of response, as it would be expected if a sensory adaptation was to occur. Nor an increase in response nor an inversion of sense of the response (i.e., meaning that there is still a response) can be explained by a sensory adaptation. Similarly, motor fatigue is discarded as a decrease in the locomotor response would also cause a lack of response.

Duration of pre-exposure specifies the sign of the non-associative cognitive modulation: short pre-exposure elicits sensitization and long pre-exposure seems to elicit habituation. Although it was not the objective of this work, we think there might be some point somewhere between *short* and *long* pre-exposure duration in which sensitization and habituation might be hidden one behind the other. It's surprising to realize that different exposure times to the same compound can generate such opposite effects. Based in our results we cannot inform whether physiological mechanisms involved in both processes are independent or not.

We show in this work that a pre-exposure to IsoAc can modulate the sensitivity to the same compound in *T. infestans* larvae by revealing changes in their escape response when confronted to different doses after a *short* or a *long* pre-exposure. However, the relevance of the dose of IsoAc used for pre-exposure was not studied. We chose here to carry out all pre-treatments by exposing larvae to a biologically relevant dose: 10 μg of IsoAc, which is similar to the quantity present in the glands, estimated by quantitative gas chromatography (Palottini, pers. communication) and elicited an innate response in our experimental setup. Further studies can be focused on analyzing if pre-exposure effect is modulated by concentration and if the switch from sensitization to habituation might be modulated not only by pre-exposure duration but also by pre-exposure concentration. Additionally, we do not know yet if pre-exposure effect is doses dependent, i.e., if pre-exposure to a certain dose only elicits a change in sensitivity to the same dose or to other also. In this work we only found a modulation of the escape response when larvae were tested to the same dose at which they were exposed.

As mentioned previously, IsoAc is the main component of the AP emitted by mechanically disturbed *T. infestans* adults (Manrique et al., 2006). Although pre-exposure to a biologically relevant dose of IsoAc increases the response of bugs to the same compound, it does not elicit a cognitive plasticity to the AP blend. The opposite is also valid, as pre-exposure to AP did not modulate the response to IsoAc. These results suggest that when an individual perceives the AP blend, it is not just the main component the responsible for the escape response, but instead a combination with other volatile compounds released during disturbance. However, it might occur that IsoAc alone is perceived as a complete different chemical entity when presented in a background of other compounds, as it is the case when the AP is perceived by these bugs. Further studies are necessary to analyze this question.

IsoAc *per se* is innately perceived by *T. infestans* larvae as an aversive stimulus. Applying classical conditioning protocols, the negative hedonic value of IsoAc was increased by associating it with a second aversive stimulus: a mechanical disturbance. Mechanical disturbance was applied as aversive stimulus as it can mimic the reaction to a predator or a defensive host bothered by triatomines. For this reason, the increase in the escape response of larvae after the co-occurrence of the contingency IsoAc/mechanical disturbance shows how the correct association between these two stimuli might be relevant for survival. Larvae might in this way increase the negative hedonic value of the AP if a potential danger becomes more real (i.e., sensing a more direct risk indicator as a mechanical vibration).

In the operant experiments, we tested if the delivery of IsoAc could act as punishment for triatomine larvae. To our knowledge, this is the first report in which an aversive odor is used as negative reinforcement in animals. IsoAc *per se*, although perceived as an aversive stimulus, is not the real danger but instead the warning of the presence of a potential danger (e.g., predator or defensive host). In these experiments larvae learned to avoid zones with higher presence of IsoAc. This learned behavior might allow triatomines to avoid zones where previously high concentrations of IsoAc were present, i.e., zones with higher probabilities to find a potential danger.

For both associative protocols applied here (i.e., classical and operant conditioning), higher concentrations of the chemical stimulus seemed to better consolidate the association presented. It is well described that salience of stimuli is a key parameter for a better memory consolidation in many animals (Pelz et al., 1997). Here, even if better escape responses were obtained by loading 10 μg of IsoAc in the stimulus piece of paper, the best memory scores were obtained when 1000 μg of IsoAc were used for both, classical and operant conditioning protocols.

We show in this report that a particular compound, IsoAc, can attain different roles in the cognitive modulation of a particular behavior. First of all, IsoAc may act as an unconditioned stimulus (US), as it generates an innate escape response (i.e., an unconditioned response) in these insects. We showed also, in Experiment 1, that IsoAc can act as sensitization agent when delivered in short puffs. Moreover, a long pre-exposure to this molecule can even inverse its biological significance for triatomines, suggesting and habituation process involved. Additionally, in Experiment 2 we show that IsoAc can act as conditioned stimulus (CS) under a classical paradigm. Finally, in Experiment 3 IsoAc takes the role of a negative reinforcement in an operant conditioning. As far as we know this is the first work in which the existence of a single molecule having so many and different cognitive roles is reported.

### Conflict of interest statement

The authors declare that the research was conducted in the absence of any commercial or financial relationships that could be construed as a potential conflict of interest.

## References

[B1] Aggio J. Rakitin A. Maldonado H. (1996). Serotonin-induced short- and long-term sensitization in the crab *Chasmagnathus*. Pharmacol. Biochem. Behav. 53, 441–448 10.1016/0091-3057(95)02015-2 8808156

[B2] Alonso W. J. Wyatt T. D. Kelly D. W. (2003). Are vectors able to learn about their hosts. A case study with *Aedes aegypti* mosquitoes. Mem. Inst. Oswaldo Cruz 98, 665–672 10.1590/S0074-02762003000500014 12973535

[B3] Anderson P. Hansson B. S. Nilsson U. Han Q. Sjoholm M. Skals N. (2007). Increased behavioral and neuronal sensitivity to sex phero-mone after brief odor experience in a moth. Chem. Senses 32, 483–491 10.1093/chemse/bjm017 17510089

[B4] Anderson P. Sadek M. M. Hansson B. S. (2003). Pre-exposure modulates attraction to sex pheromone in a moth. Chem. Senses 28, 285–291 10.1093/chemse/28.4.285 12771015

[B5] Anton S. Evengaard K. Barrozo R. B. Anderson P. Skals N. (2011). Brief predator sound exposure elicits behavioral and neuronal long-term sensitization in the olfactory system of an insect. Proc. Natl. Acad. Sci. U.S.A. 108, 3401–3405 10.1073/pnas.1008840108 21300865 PMC3044404

[B6] Bitterman M. E. Menzel R. Fietz A. Schafer S. (1983). Classical conditioning of proboscis extension in honeybees (*Apis mellifera*). J. Comp. Psychol. 97, 107–119 10.1037/0735-7036.97.2.107 6872507

[B7] Braun G. Bicker G. (1992). Habituation of an appetitive reflex in the honeybee. J. Neurophysiol. 67, 588–598 1578245 10.1152/jn.1992.67.3.588

[B8] Brindley M. D. H. (1930). On the metasternal scent-glands of certain Heteroptera. Trans. R. Entomol. Soc. Lond. 78, 199–208 10.1111/j.1365-2311.1930.tb00383.x

[B9] Cork A. Park K. C. (1996). Identification of electrophysiologically-active compounds for the malaria mosquito, *Anopheles gambiae*, in human sweat extracts. Med. Vet. Entomol. 10, 269–276 10.1111/j.1365-2915.1996.tb00742.x 8887339

[B10] Crespo J. G. Manrique G. (2007). Mating behavior of the hematophagous bug *Triatoma infestans*: role of brindley's and metasternal glands. J. Insect Physiol. 53, 708–714 10.1016/j.jinsphys.2007.03.014 17509610

[B11] Cruz-López L. Morgan E. D. Ondarza R. N. (1995). Brindley's gland exocrine products of *Triatoma infestans*. Med. Vet. Entomol. 9, 403–406 10.1111/j.1365-2915.1995.tb00013.x 8541592

[B12] Duerr J. S. Quinn W. G. (1982). Three Drosophila mutations that block associative learning also affect habituation and sensitization. Proc. Natl. Acad. Sci. U.S.A. 79, 3646–3650 10.1073/pnas.79.11.3646 6808513 PMC346480

[B13] Grubb M. S. Thompson I. D. (2004). The influence of early experience on the development of sensory systems. Curr. Opin. Neurobiol. 14, 503–512 10.1016/j.conb.2004.06.006 15321072

[B14] Guerenstein P. G. Guerin P. M. (2001). Olfactory and behavioural responses of the blood-sucking bug *Triatoma infestans* to odours of vertebrate hosts. J. Exp. Biol. 204, 585–597 11171309 10.1242/jeb.204.3.585

[B15] Guerrieri F. Gemeno C. Monsempes C. Anton S. Jacquin-Joly E. Lucas P. (2012). Experience-dependent modulation of antennal sensitivity and input to antennal lobes in male moths (*Spodoptera littoralis*) pre-exposed to sex pheromone. J. Exp. Biol. 215, 2334–2341 10.1242/jeb.060988 22675195

[B16] Hammer M. Braun G. Mauelshagen J. (1994). Food-induced arousal and non-associative learning in honeybees: dependence of sensitization on the application site and duration of food stimulation. Behav. Neural Biol. 62, 210–223 10.1016/S0163-1047(05)80019-6 7857243

[B17] Heisenberg M. Borst A. Wagner S. Byers D. (1985). *Drosophila* mushroom body mutants are deficient in olfactory learning. J. Neurogenet. 2, 1–30 10.3109/01677068509100140 4020527

[B18] Kälin M. Barrett F. M. (1975). Observations on the anatomy, histology, release site, and function of Brindley's glands in the blood-sucking bug, *Rhodnius prolixus* (Heteroptera: Reduviidae). Ann. Entomol. Soc. Am. 68, 126–134

[B19] Kaur J. S. Lai Y. L. Giger A. D. (2003). Learning and memory in the mosquito *Aedes aegypti* shown by conditioning against oviposition deterrence. Med. Vet. Entomol. 17, 457–460 10.1111/j.1365-2915.2003.00455.x 14651662

[B20] Lazzari C. R. (1992). Circadian organization of locomotion activity in the haematophagous bug *Triatoma infestans*. J. Insect Physiol. 38, 895–903 10.1016/0022-1910(92)90101-I

[B21] Manrique G. Lorenzo M. G. (2012). The sexual behavior of Chagas' Disease vectors: chemical signals mediating communication between male and female Triatomine bugs. Psyche 2012, 8 10.1155/2012/862891

[B22] Manrique G. Vitta A. Ferreira R. Zani C. Unelius C. Lazzari C. (2006). Chemical communication in Chagas Disease vectors. Source, identity, and potential function of volatiles released by the Metasternal and Brindley's glands of *Triatoma infestans* adults. J. Chem. Ecol. 32, 2035–2052 10.1007/s10886-006-9127-7 16902820

[B23] McCall P. J. Eaton G. (2001). Olfactory memory in the mosquito *Culex quinquefasciatus*. Med. Vet. Entomol. 15, 197–203 10.1046/j.0269-283x.2001.00304.x 11434554

[B24] McCall P. J. Kelly D. W. (2002). Learning and memory in disease vectors. Trends Parasitol. 18, 429–433 10.1016/S1471-4922(02)02370-X 12377586

[B25] McCall P. J. Mosha F. W. Njunwa K. J. Sherlock K. (2001). Evidence for memorized site-fidelity in *Anopheles arabiensis*. Trans. R. Soc. Trop. Med. Hyg. 95, 587–590 10.1016/S0035-9203(01)90087-2 11816426

[B26] Menzel R. Muller U. (1996). Learning and memory in honeybees: from behavior to neural substrates. Annu. Rev. Neurosci. 19, 379–404 10.1146/annurev.ne.19.030196.002115 8833448

[B27] Minoli S. Kauer I. Colson V. Party V. Renou M. Anderson P. (2012). Brief exposure to sensory cues elicits stimulus-nonspecific general sensitization in an insect. PLoS ONE 7:e34141 10.1371/journal.pone.0034141 22457821 PMC3311575

[B28] Monteith L. G. (1963). Habituation and associative learning in *Drino bohemica Mesn*. (Diptera: Tachinidae). Can. Entomol. 95, 418–426 10.4039/Ent95418-4

[B29] Pavlov I. P. (1927). Lectures on Conditioned Reflexes. New York, NY: International Publishers

[B30] Pelz C. Gerber B. Menzel R. (1997). Odorant intensity as a determinant for olfactory conditioning in honeybees: roles in discrimination, overshadowing and memory consolidation. J. Exp. Biol. 200, 837–847 9076967 10.1242/jeb.200.4.837

[B31] Pontes G. Bohman B. Unelius C. R. Lorenzo M. (2008). Metasternal gland volatiles and sexual communication in the triatomine bug, *Rhodnius prolixus*. J. Chem. Ecol. 34, 450–457 10.1007/s10886-008-9431-5 18317844

[B32] Pontes G. Lorenzo M. G. (2012). Female metasternal gland odors mediate male aggregation in *Rhodnius prolixus*, a triatomid bug. Med. Vet. Entomol. 26, 33–36 10.1111/j.1365-2915.2011.00983.x 22077398

[B33] Rakitin A. Tomsic D. Maldonado H. (1991). Habituation and sensitization to an electrical shock in the crab *Chasmagnathus*. Effect of background illumination. Physiol. Behav. 50, 477–487 10.1016/0031-9384(91)90533-T 1800998

[B34] Rojas J. C. Rios-Candelaria E. Cruz-Lopez L. Santiesteban A. Bond-Compean J. G. Brindis Y. (2002). A reinvestigation of Brindley's gland exocrine compounds of *Rhodnius prolixus* (Hemiptera: Reduviidae). J. Med. Entomol. 39, 256–265 10.1603/0022-2585-39.2.256 11931024

[B35] Schacter D. L. Buckner R. L. (1998). Priming and the Brain. Neuron 20, 185–195 10.1016/S0896-6273(00)80448-1 9491981

[B36] Schofield C. Upton C. P. (1978). Brindley's scent-glands and the metasternal scent-glands of *Panstrongylus megistus* (Hemiptera, Reduviidae, Triatominae). Rev. Bras. Biol. 38, 665–678

[B37] Schofield C. J. (1979). Demonstration of isobutyric acid in some triato-mine bugs. Acta Trop. 36, 103–105 35929

[B38] Skinner B. F. (1937). Two types of conditioned reflex: a reply to Konorski and Miller. J. Gen. Psychol. 16, 272–279 10.1080/00221309.1937.9917951

[B39] Vinauger C. Buratti L. Lazzari C. R. (2011a). Learning the way to blood: first evidence of dual olfactory conditioning in a blood-sucking insect, *Rhodnius prolixus*. I. Appetitive learning. J. Exp. Biol. 214, 3032–3038 10.1242/jeb.056697 21865515

[B40] Vinauger C. Buratti L. Lazzari C. R. (2011b). Learning the way to blood: first evidence of dual olfactory conditioning in a blood-sucking insect, *Rhodnius prolixus*. II. Aversive learning. J. Exp. Biol. 214, 3039–3045 10.1242/jeb.057075 21865516

[B41] Vinauger C. Pereira M. H. Lazzari C. R. (2012). Learned host preference in a Chagas disease vector, *Rhodnius prolixus*. Acta Trop. 122, 24–28 10.1016/j.actatropica.2011.11.007 22138145

[B42] Vitta A. Bohman B. Unelius C. Lorenzo M. (2009). Behavioral and electrophysiological responses of *Triatoma brasiliensis* males to volatiles produced in the Metasternal glands of females. J. Chem. Ecol. 35, 1212–1221 10.1007/s10886-009-9709-2 19902303

[B43] Walters E. Illich P. Weeks J. Lewin M. (2001). Defensive responses of larval *Manduca sexta* and their sensitization by noxious stimuli in the laboratory and field. J. Exp. Biol. 204, 457–469 11171298 10.1242/jeb.204.3.457

[B44] Ward J. P. (1981). A comparison of the behavioural responses of the haematophagous bug, *Triatoma infestans* to synthetic homologues of two naturally occurring chemicals (n- and isobutyric acid). Physiol. Entomol. 6, 325–329 10.1111/j.1365-3032.1981.tb00277.x

[B45] Zacharias C. Pontes G. Lorenzo M. Manrique G. (2010). Flight initiation by male *Rhodnius prolixus* is promoted by female odors. J. Chem. Ecol. 36, 449–451 10.1007/s10886-010-9779-1 20352301

